# Softwares and methods for estimating genetic ancestry in human populations

**DOI:** 10.1186/1479-7364-7-1

**Published:** 2013-01-05

**Authors:** Yushi Liu, Toru Nyunoya, Shuguang Leng, Steven A Belinsky, Yohannes Tesfaigzi, Shannon Bruse

**Affiliations:** 1 Lovelace Respiratory Research Institute, 2425 Ridgecrest Dr. S.E, Albuquerque, NM, 87108, USA; 2 VA Medical Center, Albuquerque, NM, USA

**Keywords:** Ancestry, Genetic, Polymorphism, Structure

## Abstract

The estimation of genetic ancestry in human populations has important applications in medical genetic studies. Genetic ancestry is used to control for population stratification in genetic association studies, and is used to understand the genetic basis for ethnic differences in disease susceptibility. In this review, we present an overview of genetic ancestry estimation in human disease studies, followed by a review of popular softwares and methods used for this estimation.

## Introduction

The analysis of population structure based on genetic ancestry is an increasingly important component of many genetic studies. Genetic ancestry estimation is a broad term which is concerned with a number of different population genetics problems, including: (1) detection of population structure (2) defining the number of subpopulations in a sample (3) assigning individuals to subpopulations (4) defining the number of ancestral populations in admixed populations (5) assigning ancestral population proportions to admixed individuals and (6) identifying the genetic ancestry of distinct chromosomal segments within an individual [1]. No single method or software can optimally solve all of these problems. This review will present a number of softwares for defining these various facets of genetic ancestry, with an emphasis on their use in medical genetic studies.

Genetic ancestry arose from the biogeographical distribution of human populations, and is a concept distinct from ethnicity, which is a social construct with no clear genetic definition [2]. The establishment of inexpensive single nucleotide polymorphism (SNP) genotyping platforms in the previous decade has allowed for relatively facile collection of markers to assess genetic ancestry in human populations. With prior knowledge of population-specific allele frequencies, panels of SNPs referred to as ancestry informative markers (AIMs) can be used to estimate genetic ancestry [3-6]. AIMs are markers whose frequencies are significantly different, and thus able to distinguish, between two or more populations [7]. Panels of AIMs vary in size, depending on the intended purpose. Relatively small panels numbering in the dozens to hundreds of SNPs can be used when the purpose is to define continental genetic ancestry, whereas hundreds or thousands of SNPs are required for more refined sub continental estimation or for traditional mapping by admixture linkage disequilibrium (MALD) [8-10]. Alternatively, the advent of genome-wide association studies (GWAS) has made it increasingly common to use the large amount of SNP data already present on genome-wide arrays to estimate genetic ancestry [11]. Some of the methods presented in this review do not require the use of specific AIM panels, but work more effectively with dense genotyping data, though different softwares are more or less adept at handling different sized marker sets. Subsets of AIMs can also be selected from GWAS data using traditional approaches based on SNP informativeness [12] or more recent principal components analysis (PCA) approaches [13].

In medical genetics, perhaps the most common use of estimated genetic ancestry is as a control against cryptic population stratification in genetic association studies [8,14]. Confounding by population stratification can occur when (a) the allele frequencies of a disease causing SNP are substantially different between populations (b) population proportions are not matched in cases and controls (c) population structure isn’t accounted for in the statistical modeling of genetic association. Self-identified ethnicity can be used to control for this potential confounding, often by simply including individual ethnicity as a covariate in the regression models or by performing population stratified analyses. However, using self-identified ethnicity can result in misclassification [15], and also cannot account for varying degrees of admixture within self-identified ethnic groups. Direct estimates of genetic ancestry based on genotype data are therefore preferred as a control for population substructure, given the advantage in precision and informativeness over self-identified ethnicity [16]. In addition to serving as a control for population stratification, estimation of genetic ancestry has become particularly important in studies of recently admixed populations, such as African-Americans and Latinos [3,17]. Admixed populations which show protection or susceptibility to common diseases provide an unprecedented opportunity for disease gene mapping [18,19].

Genetic ancestry can be divided into “local” and “global” estimates [20]. Local estimates are concerned with identifying the ancestral origin of distinct chromosomal segments within an individual genome, and these methods are a more recent development in the field. Global estimates seek to establish ancestral proportions averaged across the genome of an individual, so that proportions of each ancestry (summing to 1) can be assigned to each individual. In general, the softwares for estimating genetic ancestry can also be divided into methods that rely on multivariate statistical methods (like PCA and cluster analysis) versus methods that make use of explicit genetic models, although this distinction does not imply that there aren’t important similarities between algorithmic and model-based methods. The purpose of this survey is to describe some of the better known algorithmic and model-based programs for estimating both local and global genetic ancestry. Table 1 contains a list of the softwares under discussion, usefulness in estimating global or local ancestry, the computing environment, and a link to software website. All softwares presented in this review are free to academic users.

**Table 1:** Softwares for estimating genetic ancestry

**Software**	**Global**/**local estimation**	**Operating Environments**	**Link**
**STRUCTURE**	Global	Windows/DOS/Linux/Solaris/Mac	http://pritch.bsd.uchicago.edu/structure.html
***frappe***	Global	Windows/Linux/Mac	http://med.stanford.edu/tanglab/software/frappe.html
**ADMIXTURE**	Global	Linux/Mac	http://www.genetics.ucla.edu/software/admixture/index.html
**EIGENSTRAT**/**smartpca**	Global	Linux	http://www.hsph.harvard.edu/faculty/alkes-price/software/
**ipPCA**/**EigenDev**	Global	Windows/Linux (MatLab)	http://www4a.biotec.or.th/GI/tools/ippca
**GEMTools**	Global	Windows/Linux	http://www.wpic.pitt.edu/wpiccompgen/GemTools/GemTools.htm
**PLINK**	Global	Windows/Linux/Mac/C/C++	http://pngu.mgh.harvard.edu/~purcell/plink/
**LAMP**	Local and Global	Windows/Linux	http://lamp.icsi.berkeley.edu/lamp/
**SABER**	Local and Global	Linux	http://med.stanford.edu/tanglab/software/saber.html
**HAPMIX**	Local and Global	Unix/Linux/Windows	http://www.stats.ox.ac.uk/~myers/software.html
**ANCESTRYMAP**	Local and Global	Unix/Linux	http://genepath.med.harvard.edu/~reich/Software.htm

### Estimating global ancestry

#### Structure

STRUCTURE, perhaps the most widely used program for estimating global genetic ancestry, was developed by Pritchard et. al. in 2000 [1]. STRUCTURE is a model-based clustering approach which utilizes genotype data to infer the presence of distinct populations, assign individuals to populations, identify admixture proportions at the individual level, and to estimate ancestral population allele frequencies in admixed populations. There are four main models within STRUCTURE: (1) No admixture model, which assumes individuals come from distinct populations (2) admixture model (3) linkage model [21], which accounts for admixture linkage disequilibrium (i.e. the phenomenon whereby recently admixed populations have larger regions of LD between loci), and (4) prior population information models, which can use location or self-identified ethnicity (if they are informative) to enhance the detection of population structure [22]. The model which utilizes prior population information is particularly useful when working with a small number of markers or when population structure is weak.

Prior to running STRUCTURE, parameters must be set, with perhaps the most critical being K, the number of populations. The authors took an *ad hoc* approach for K, estimating the conditional mean and variance of Bayesian deviance based on the data [1]. It is also useful to consider known information on the populations under study when choosing an appropriate K. As with any method used to estimate global ancestry proportions, a highly informative marker set and better representation of ancestral populations allows for more exquisite resolution of population structure, and thus will influence the selection of K. In order to run STRUCTURE, individual genotypes are required as input. For the linkage model, phase and genetic map data can also be used.

Briefly, STRUCTURE models the probability of observed genotypes given the individual ancestry proportions and ancestral population allele frequencies. The program assigns individuals to ancestral populations (or proportions of individuals in the case of the admixture model) based on genotype data, while simultaneously estimating the allele frequencies of those populations. Given prior information about the probability of the populations of origin for individuals and the probability of allele frequencies for all populations, traditional Bayesian methods using Markov chain Monte Carlo (MCMC) and Gibbs Sampling are used to obtain the corresponding posterior distribution [1]. A burn-in period is required to reach a stationary posterior distribution, and this burn-in period (and the number of MCMC iterations) is set by the user. MCMC methods are useful in obtaining samples from a posterior distribution when direct sampling is not possible. The method utilizes the Metropolis-Hastings algorithm to obtain a sequence of random samplings which can approximate the distribution.

#### Admixture

Similar to STRUCTURE, the ADMIXTURE program models the probability of observed genotypes using ancestry proportions and population allele frequencies, simultaneously estimating population allele frequencies along with ancestry proportions. An input file of genotypes from unrelated individuals is required, as is an estimate of K. The ADMIXTURE program uses a cross-validation approach to help estimate K, unlike STRUCTURE which computes the model evidence for each value of K. The ADMIXTURE cross validation procedure helps identify which value of K has the best predictive value, by masking or holding out a subset of genotype data and then predicting those masked genotypes.

Unlike STRUCTURE, ADMIXTURE focuses on maximum likelihood estimation (MLE) rather than sampling the posterior distribution using MCMC, and calculates the estimates via a block relaxation approach which results in improvements in speed [20,23]. This computational efficiency provides an advantage over STRUCTURE when using very large numbers of markers, for example when using dense GWAS data instead of smaller AIM panels. Briefly, ADMIXTURE updates the allele frequency parameter and ancestry fraction parameter alternatively by maximizing the second-order Taylor’s expansion of the likelihood function. It does this iteratively, based on allele frequencies and ancestry proportions associated with the current parameter values. This is typically known as sequential quadratic programming and coincides with Newton’s method in the absence of constraints [24]. Newton’s method can be used to find the optimal point to solve x-M(x) =0. However, obtaining the differential of M(x) is challenging; therefore a quasi-Newton method is used. This accelerates the convergence, and has been shown to provide an advantage in speed over convergence methods like the Expectation Maximization (EM) algorithm, as employed in the MLE-based program *frappe* (discussed below). Alexander and coworkers showed that on real world datasets, ADMIXTURE is much faster than STRUCTURE but with comparable estimation, and has been shown to be faster and more accurate than *frappe*[20].

##### Frappe

*Frappe* uses a full maximum likelihood approach to estimate individual admixture [25]. *frappe* requires a genotype and parameter file, containing individual genotypes and a specification of K. Unlike STRUCTURE and ADMIXTURE, *frappe* does not provide measures to choose an optimal K value. *frappe* is far more computationally efficient than STRUCTURE [25], but as stated above, less computationally efficient than ADMIXTURE. In simulations using few SNPs (n=60), few individuals from ancestral populations (n=20 and n=60), and low information content of the SNPs (average delta=0.33), *frappe* produced significantly less biased estimates than STRUCTURE [25]. Thus, *frappe* appears to perform well when population structure is weak. However, STRUCTURE can use population information to inform the prior probabilities, and this model is also effective in the case of weak population structure [22].

#### Eigenstrat and ipPCA

PCA can be used for dimensionality reduction to group those with similar genetic ancestry together [26]. PCA is a computationally efficient method which can handle large numbers of markers, and is useful for visualizing population structure [27,28]. The first few principal components are often used to correct for population stratification in genetic association studies. The EIGENSOFT software package contains EIGENSTRAT (and its helper routine smartpca), and is the most cited PCA method for population structure applications [29].

Briefly, the PCA methods focus on the spectral decomposition of a variance covariance matrix for dimensionality reduction. Both the eigenvalues and eigenvectors are important for underlying population structure identification. The eigenvectors present the linear combination of the covariates which in turn serve as the new dimensions. All the dimensions are orthogonal to each other. These linear combinations are known as the principal components. If there is underlying structure among populations, PCA tends to separate them based on the principal components. A question, however, is when to stop dividing individuals into subpopulations. Patterson and colleagues provided an answer which allows determination of the probability of structure based on the Tracy-Widom distribution [29]. The Tracy-Widom theory considers that when the dimension for a matrix M is suitably large, the distribution of the largest eigenvalue follows approximately the Tracy Widom distribution as identified by Johnstone [30]. This allows assessment of the probability that the largest eigenvalue is random, and thus, whether or not structure exists [29].

In large, highly structured samples, particularly when subpopulations are closely related or when there is a genetically distant subpopulation, traditional PCA methods have difficulty assigning individuals to the correct subpopulation [31]. However, ipPCA (and an extension termed EigenDev-ipPCA) is a refinement of the PCA method which efficiently assigns individuals to populations and provides accurate estimates of K, even in highly structured populations [31,32]. Although there is no general agreement on what constitutes a subpopulation, on simulated and real datasets, ipPCA was more accurate (ie. fit better with population assignments based on prior knowledge of population structure) than STRUCTURE in determining K, particularly as the number of subpopulations increased [31].

#### Plink and GEMTools

Other algorithmic approaches statistically related to the PCA exist as well. PLINK implements multidimensional scaling (MDS) to assess population structure. Given that PLINK is commonly used for genetic association testing, it is convenient that the output file from MDS analysis in PLINK can be directly used as a covariate file in PLINK-based association testing. In the current version of PLINK, genome-wide coverage of SNPs is required to perform the MDS analysis. MDS is a class of statistical analysis that provides a view of the proximities for objects. Therefore, the similarities of people based on genetic ancestry can be viewed using MDS. Theoretically, the MDS method tries to find a matrix from the dissimilarity matrix that preserves the distances, allowing the data to be projected into low dimensional space [33]. PLINK utilizes a distance measure based on genome-wide pairwise IBS (identity-by-state) to construct an MDS plot. In a comparative study, PCA structure analysis as implemented in EIGENSOFT performed slightly better than PLINK-based MDS analysis in correcting for population stratification in a GWAS dataset [34].

Another algorithmic approach is a recently introduced package called GEMTools which uses spectral graph theory for dimensionality reduction and clustering by genetic ancestry [35]. This approach may be more flexible than PCA [36], and the package contains a convenient function for matching cases and controls based on genetic similarity.

### Estimating local ancestry

#### Lamp

LAMP (Local Ancestry in adMixed Populations) is a program used to infer locus-specific ancestry in admixed populations using sliding windows of contiguous SNPs [37,38]. A significant advantage of LAMP relative to other methods for local ancestry is that it does not require genotypes from unadmixed ancestral populations as input. This is advantageous when working with an uncharacterized populations or when ancestral genotypes aren’t available. When available, ancestral genotype information can be utilized by the LAMP program using LAMP-ANC. The LAMP-ANC program then infers local ancestry based on the ancestral populations, rather than the de novo inference used by LAMP.

Similar to other local ancestry programs, LAMP does require input parameters, including the recombination rate, global ancestry proportion, and an upper limit on time since admixture. Global recombination rates have previously been calculated [39], and global ancestral proportion can be calculated using a program such as STRUCTURE. Simulations indicate that LAMP is more robust to inaccuracies in time since admixture and less so to inaccuracies in global ancestral proportions, and performs somewhat less well as this proportion nears 0.5 [37]. On simulated admixed populations representing African-Americans, LAMP and LAMP-ANC were on average more accurate and considerably faster than SABER (discussed below) [37]. However on simulations of admixed populations with very closely related ancestral populations (Chinese and Japanese), SABER was more accurate than LAMP but less accurate than LAMP-ANC. However, all methods perform rather poorly when the ancestral populations are very closely related. All of the local ancestry methods can be used to estimate global ancestry by chromosomal or genome-wide averaging of the local ancestry estimates. On simulated data, relative to STRUCTURE, LAMP was more accurate in estimating global genetic ancestry [37]. Again on simulated data, LAMP is capable of accurately estimating admixture proportions in cases of three-way, and presumably greater, admixture. In contrast to SABER and HAPMIX (reviewed in following sections), LAMP does not model LD and assumes uncorrelated SNPs, though the program is somewhat robust to this assumption [37].

Briefly, the idea of LAMP is to select a suitable window length, and then a clustering algorithm known as Iterated Conditional Modes (ICM) is used to estimate the likelihood that an individual chromosome has a particular ancestry within this window. The ancestry of individual SNPs is determined by majority vote using all such overlapping windows containing that SNP [37]. For this procedure, the most important step is the Iterated Conditional Modes. This algorithm differs from the traditional Expectation Maximization (EM) algorithm in the E step [37]. In the EM algorithm, the expected classification based on the minor allele frequencies of the SNPs within the given window will be obtained; in contrast, in the ICM algorithm, the maximized posterior estimate for the classification based on the minor allele frequencies and genotype will be obtained instead, assuming the initial classification is reasonable [37]. Therefore, ICM can have a greatly accelerated convergence compared to the EM algorithm. Since this algorithm involves the accurate estimation of the minor allele frequency as the starting point, the authors of this software considered two scenarios: (1) in the case of two ancestral populations with unknown allele frequencies in the ancestral population, the MAXVAR algorithm will be used, in which the individuals will be grouped according to the measurement of similarity (2) in the case when there are two or more ancestral populations and the minor allele frequency is known, the question is simpler and the given ancestral allele frequencies will be used [37].

#### Hapmix

HAPMIX is an extension of a Hidden Markov Model (HMM) [40] used to model linkage disequilibrium in population genetic data [41]. HAPMIX requires as input phased data from ancestral populations, unphased data from the admixed population, and a recombination rate file which give the physical and genetic position (in cM) of each SNP. Like LAMP and SABER, HAPMIX is used to determine genetic ancestry for each chromosomal position or segment in the genome. Unlike those other programs, HAPMIX makes use of haplotype information. This requires the use of phased genotype data from unadmixed ancestral populations, and the current version of HAPMIX can only handle two-way admixture.

In admixed populations, linkage disequilibrium exists at a coarse scale and fine scale [41]. Course scale admixture linkage disequilibrium is due to relatively recent recombination events which result in individual genomes being comprised of distinct chromosomal segments inherited from particular ancestral populations. Fine scale linkage disequilibrium is based on historical recombination events in the ancestral populations. Modeling of both, using a program such as HAPMIX, may increase the power of genetic association testing [41], as demonstrated in a recent study of breast cancer in African-American women [11]. Full modeling of the ancestral LD may also lead to more accurate estimates of local genetic ancestry, as demonstrated using simulated and real world data of African-Americans, where HAPMIX outperformed both ANCESTRYMAP and LAMP-ANC [41]. Further simulations demonstrated that the HAPMIX performance advantage increased with increasing time since admixture, indicating its utility across a range of admixed populations [41].

Briefly, in HAPMIX the haplotype of an individual is viewed as a mosaic of the haplotypes from the ancestral populations. At each position in the genome the likelihood that the haplotype arises from a particular ancestral population is estimated, and a Hidden Markov Model combines these likelihoods with information from neighboring loci to give probabilistic evidence that particular segments come from one ancestral population versus another [41]. Importantly, HAPMIX treats the ancestral population as unambiguously phased, but uses a built in phasing algorithm on the admixed population and doesn’t assume that any one haplotype phasing is correct. This flexible approach can help avoid inappropriate inferences of ancestry transitions [41]. Additional advantages of HAPMIX are very accurate inferences of date of admixture and the ability to accurately estimate 0, 1, or 2 ancestral alleles at each locus [41].

#### Saber

SABER is a program suitable for genome-scale data which uses a “Markov-hidden Markov model” to estimate local ancestry [42]. This local ancestry is referred to as “ancestral blocks”. Like HAPMIX, it models the ancestral LD; however it does not model haplotype structure. Input files are typical and include genotype data from ancestral and admixed individuals, global ancestry estimates of admixed individuals, and physical map location of the SNPs. In addition to providing localized ancestry (with graphical output), SABER can be used to estimate time since admixture.

#### Ancestrymap, admixmap, and maldsoft

A number of other methods can infer local (and global) ancestry, but are not computationally efficient when working with genome-scale data. These include ANCESTRYMAP [43], ADMIXMAP [44], and MALDSOFT [45]. These are all well-established methods which use Hidden Markov Models to combine data across loci to infer ancestry at each locus, and these programs require that there be no LD between markers. The primary focus of these programs is for traditional admixture mapping on AIM panels, and not the evaluation of local genetic ancestry using dense panels of markers [25].

## Conclusion

The programs presented here offer tools to deal with a number of population genetics problems related to genetic ancestry. No single program is sufficient for dealing with the variety of research questions being asked, and using combinations of these programs may be most helpful for the next generation of medical genetics studies. For example, while global ancestry has historically been used to control for population stratification in association studies, it may be more appropriate to control for both local and global ancestry [46], which may be optimally resolved using separate programs. New uses for these programs are also arising. Several recent studies have assessed the correlation of individual ancestry proportions with disease risk or treatment response [47-50]. For example, it was reported that the percent Native American genetic ancestry in a cohort of children was associated with risk for relapse after chemotherapeutic treatment of acute lymphoblastic leukemia [49]. Understanding why ancestry proportions in admixed populations correlate with phenotypes will require precise identification of the ancestry specific loci that are responsible. Recent reports have demonstrated that statistical tests combining admixture and ancestral linkage disequilibrium signals is a more powerful method of testing for genetic association than MALD or traditional LD mapping individually [11,41]. Genetic ancestry softwares which give refined and accurate estimates of local ancestry are critically important to this next generation of genetic studies in admixed populations.

## Competing interests

We declare that we have no competing interests.

## Authors’ contribution

YL and SB made substantial contributions to conception and design of the manuscript, and interpretation of statistical methods. YL, TN, SL, SAB, YT, and SB made substantial contributions to drafting the article or revising it critically for important intellectual content. All authors read and approved the final manuscript.
